# Reservation wage of female volunteer community health workers in Dhaka urban slums: a bidding game approach

**DOI:** 10.1186/s13561-014-0016-4

**Published:** 2014-09-05

**Authors:** Khurshid Alam, Sakiba Tasneem, Molla Huq

**Affiliations:** Equity and Health Systems, International Centre for Diarrhoeal Disease Research, Bangladesh (ICDDR,B), 68 Shaheed Tajuddin Ahmed Sharani, Mohakhali, Dhaka, 1212 Bangladesh; Monash School of Public Health & Preventive Medicine, Monash University, 99 Commercial Road, Level 5, The Alfred Centre, Melbourne Vic, 3004 Australia; BRAC Research & Evaluation Division, BRAC Dhaka, 1212 Bangladesh; Department of Economics, Monash University, Vic, 3800 Australia

**Keywords:** Volunteer CHWs, Performance, Retention, Financial incentives, Reservation wage, Bidding game

## Abstract

**Background:**

BRAC, a large Bangladeshi NGO, recently has been using female volunteer community health workers (CHWs) in Dhaka urban slums to provide maternal and child health services. Due to erratic performance-based income and higher opportunity cost the urban CHWs lose motivation which contributes to high dropout and poor performance. This results challenges for the cost effectiveness and sustainability of the urban health program. CHWs also consider their performance-based income very low compare to their work load. So, CHWs raise their voice for a fixed income. In order to understand this problem we explored fixed income for CHWs and the correlates that influence it. We surveyed a sample of 542 current CHWs. We used *bidding game* approach to derive the *equilibrium* reservation wage for CHWs for providing full-time services. Then, we performed ordered logit models with bootstrap simulation to identify the determinants of reservation wage.

**Results:**

The average reservation wage of CHWs to continue their work as full-time CHWs rather than volunteer CHWs was US$24.11 which was three times higher than their current performance-based average income of US$ 8.03. Those CHWs received additional health training outside BRAC were 72% and those who joined with an expectation of income were 62% more likely to ask for higher reservation wage. On the contrary, CHWs who were burdened with household loan were 65% and CHWs who had alternative income generating scope were 47% less likely to ask for higher reservation wage. Other important factors we identified were BRAC village organization membership, competition with other health services providers, performance as a CHW, and current and past monthly CHW income.

**Conclusions:**

The findings of this study are relevant to certain developing countries such as Bangladesh and Tanzania which commonly use volunteer CHWs, and where poor retention and performance is a common issue due to erratic and performance-based income. So, the study has implications in improving retention of health workers as well as their level of performance. The study also suggests that the financial incentives provided to CHWs should be clearly based on their qualifications and opportunity cost to ensure a high performing and motivated health workforce.

## Background

Globally, the crisis of health human resources is a pressing issue. It has been estimated that there is a shortage of at least four million health workers worldwide [1,2]. The typical scenario of health workforce in Bangladesh exacerbates poor provider-population ratios [3] as it does in many other low-income countries. This is, indeed, a reflection of global crisis which is considered as a major obstacle to achieving the health-related millennium development goals (MDGs) by 2015 [4,5]. Under these circumstances, community health workers (CHWs) provide a crucial and strategic solution to the shortage of skilled health workforce filling this gap in many developing countries including Bangladesh.

In response to health workforce crisis, BRAC, a large Bangladeshi NGO, has become a pioneer in the use of female volunteer CHWs popularly known as *Shasthya Shebikas* in its community based health programs since 1977. BRAC recruits and trains these CHWs who serve as the first point of contact between community members and BRAC health services [6]. Currently, 80,000 female volunteer CHWs under BRAC Health Program are serving as frontline workers at the community level. BRAC CHWs are female, aged 25–40 years, married and the youngest child must not be less than two years of age. They have reading and writing skills. BRAC usually recruits them from Village organizations (VO) members in their own community. They are willing to work on a voluntary basis and are acceptable to their community. On joining each CHW receives a three-week basic training on primary healthcare and the use of relevant drugs and BRAC health commodities. They also receive refresher training in each month.

BRAC CHWs usually provide essential healthcare services including tuberculosis treatment in rural areas. But BRAC has recently introduced these CHWs in urban slums in a maternal, newborn and child health project called *Manoshi* reaching over 6.2 million slum population as it is being used as the key approach to improving maternal and child health of the communities in many developing countries [7]. In *Manoshi* each CHW is responsible for overseeing an average of 200 households and visiting 8–10 of these per day. They visit homes to disseminate health messages, identify pregnancies, bring pregnant women to delivery centres, accompany them during their delivery and provide newborn care. For providing the above services in the community CHWs earn a modest income although they serve as volunteers [8]. But their income is performance-based and is often erratic which is the major reason of their high dropout [9] and poor performance [10].

Recently, the experience of *Manoshi* program with CHWs has highlighted two key issues: challenges of retention of CHWs and the suboptimal performance of those who remain in the program. The challenges of retention have been explored in Alam *et al. *[9] and in Alam & Oliveras [11], and performance elsewhere [10]. The impact of dropout of CHWs also has been explored elsewhere [12]. During the field work of retention and performance studies, many CHWs raised their voice that they would prefer a fixed income rather than performance-based erratic earnings. So, for understanding the issues comprehensively, the *Manoshi* program also extended its interest in exploring expected fixed income for CHWs and the associated correlates. It is worth to note that health workers significantly prefer a flat payment for their services as opposed to competitive price as it is evidenced in a discrete choice experiment in a community-based health insurance scheme in Burkina Faso [13].

Financial income has been found to be the key factor linked to CHW retention [9]. Past studies also have found expectation of income from CHW role is a major motivation behind joining this workforce [9,14,15]. It should be mentioned that even though, these health workers work on a voluntary basis, they earn some financial incentives from selling essential health commodities and medicines and providing health services. The provision of earning income from providing these health services arise a legitimate question whether this workforce can be labelled as volunteers since the definition of volunteerism always excludes the possibility of financial gain from the services provided by a volunteer [16]. Currently, performance-based financial incentives that CHWs receive from their work are also very low considering their work load. Due to low and erratic nature of their income and higher opportunity cost of their time they can’t consider CHW role as a permanent occupation. The opportunity cost of working as a CHW is even higher in urban areas than rural areas as urban areas offer a range of opportunities with higher financial income such as working for garment factories. The unstable income from CHW work sometimes lessens family support, especially from their husbands.

Due to lack of appropriate compensation, CHWs lose their motivation, which significantly contributes to their dropout and poor performance resulting challenges for the cost effectiveness and sustainability of the program. This suggests finding out a minimum amount of fixed income that ensures proper accomplishment of CHW job as well as exploring the effects of the associated factors on the fixed income level. Therefore, re-shaping of the volunteer CHW model in terms of providing them with a minimum fixed income higher than their reservation wage, a minimum wage at which a CHW would be willing to discharge her duties properly [17], might be an important way to addressing the problem of high dropout rate and poor performance. Past studies provide some indication regarding their expectation of a fixed income and frustrations over erratic income but they do not provide a rigorous assessment on their willingness to accept a fixed reservation wage for accomplishing their job and the associated correlates. This study aims to explore the reservation wages of these CHWs along with the factors affecting it applying an econometric assessment. With a view to exploring the correlates of reservation wage we perceived a set of hypotheses to test. Those volunteer CHWs have joined with an expectation of income are more likely to ask for higher reservation wage; those who have more education and training are more likely to ask for higher reservation wage; and those who perform better and earn higher income are more likely to ask for higher reservation wage. On the contrary, those volunteer CHWs have better asset holdings and/or alternative income generating scopes are less likely to ask for higher reservation wage; and those who are burdened with household loan are less likely to ask for higher reservation wage. Finally, those CHWs are facing completion from other providers in the community are less likely to ask for higher reservation wage.

## Methods

### Sample

For a case–control study on retention during the first two years of the project, a simple random sample of 542 current CHWs from the population of 1,125 current CHWs listed in the *Manoshi* registers at the time of the study was selected [9]. We simultaneously designed the present study within the original case–control study on retention. The controls from that case–control study, 542 current CHWs (that is, retained), formed the sample for this present study.

For the case–control study on retention, the World Health Organization’s Epi Info software (http://www.who.int/chp/steps/resources/EpiInfo/en/) was used to estimate the required sample size assuming an unmatched case–control design. The study estimated the proportion of controls who were exposed to factors of interest (difficulty in educating children and fears and misconceptions of family members about BRAC health program) at 15%, based on an existing study of BRAC urban CHWs [18]. In addition, the study assumed that the odds ratio of dropout associated with exposure was 2 at the 95% confidence level and 80% power. For a ratio of one case to four controls, the number of cases required was at least 133 dropout CHWs. The study sampled both current CHWs and dropout CHWs using project data and ended up with a total sample of 146 dropout CHWs and 542 current CHWs who participated in the survey. The detailed sampling strategy was also described elsewhere [9,10].

Using a structured questionnaire we collected data from 542 current CHWs on socio-demographic characteristics, household assets, current CHW activities, income, family attitude, community approval, competition and so on after having informed consent from each CHW. Finally, we asked them whether a fixed income would encourage them to discharge their CHW role properly. We found 94% of them confirmed that they would be willing to discharge CHW activities properly if they would have been provided with a monthly fixed income. Thus our final sample consisted of 510 current CHWs and we attempted to find the reservation wage for them so that they would ensure their job well-done. The Institutional Review Board of International Centre for Diarrheal Disease Research, Bangladesh (ICDDR,B) approved this study.

### Bidding game approach

People usually assume a bargaining stance when they are asked about their reservation wage. Thus the wage they report as their reservation wage is the one that they normally start bargaining with and is above their minimum acceptable wage [19]. One study addressed this problem suggesting that the interviewer should “read each salary or wage bracket starting from the lowest and identify the first wage or salary bracket that the respondent would be willing to accept” [20]. In our study we applied *bidding game* technique [21] to determine their willingness to accept a fixed income assuming that it would minimize the possibility of overstating the reservation wage. Before stating bidding, we provided them with the following precise description of terms and conditions of the revised job offer of a CHW:

- A CHW would be paid a monthly fixed income for her services at BRAC
- She would be required to perform all the duties she is currently performing as a CHW and a list of responsibilities would be provided
- Her total working hours and holidays would be equal to that of a *Shasthya Karmi* (her immediate senior worker who work as a full-time worker)
- She would not receive any extra financial incentives beyond her fixed income for providing health services or selling health commodities; however, she would be still required to perform these services as part of her CHW responsibilities
- Her work would be more closely monitored, and
- She would not have been paid if she would not perform her assigned tasks

Thus we offered them a fixed income per month for working as a CHW following the above conditions. When they agreed with the mentioned income level, we lowered it down and down until they disagreed with our offer. On the other hand, when they disagreed at the first place, we increased the amount until they agreed with the offered amount. The maximum amount we offered them was Bangladeshi Taka (BDT) 1800 (=US$ 26.66) per month as it was the amount their immediate senior full-time worker, *Shasthya Karmi* received per month at the time of data collection of this study. If someone was not still happy with the amount we offered, we asked them to mention their minimum price for their CHW services. The minimum income we offered them was BDT 100 (US$ 1.48) as it was reported in a past study as the minimum monthly income of a volunteer CHW [14]. Thus from the bidding process, we reached to an *equilibrium* amount of fixed income which reflects a CHW’s reservation wage for duly furnishing her job. We used four different starting amounts of fixed income, i.e., BDT 500 (US$ 7.41), BDT 600 (US$ 8.89), BDT 700 (US$ 10.37) and BDT 800 (US$ 11.85) to reduce the problem of s*tarting point bias.* In bidding games, the starting point given to respondents can influence the final bid offered. This can be caused by impatience of the respondent or can happen because a starting point may suggest what size of a bid is appropriate [22]. The study also took care of *strategic bias* so that CHWs could not inflate their reservation wage.

### Econometric model

In this paper we estimated an explanatory model for the fixed income of CHWs as full-time workers. We divided the fixed income into three categories of ordinal nature assuming these three categories attribute three levels of performance: inactive, moderately active and active CHWs respectively [10]. With these three categories the dependent variable y (fixed income) can take values between 0 and 2.

The ordered logit model [23] is based on the following specification:

$$ y*\kern0.5em =\kern0.5em {\displaystyle \sum_{k=1}^K{\beta}_k{X}_k+\varepsilon \kern0.5em =\kern0.5em Z+\varepsilon } $$

where y* is the exact but unobserved dependent variable which determines the values of the observed variable *y*; *x* is the vector of independent variables, and *β* is the vector of regression coefficients which we wish to estimate and *ε* is disturbance term. Further suppose that while we cannot observe y*, we, instead, can only observe the categories of response variable *y* where it is observed when the unobserved variable y* have crossed a particular threshold *μ*. Here, if we have 2 thresholds, then

$$ y\kern0.5em =\kern0.5em \left\{\begin{array}{c}\hfill 0\hfill \\ {}\hfill 1\hfill \\ {}\hfill 2\hfill \end{array}\right.\begin{array}{c}\hfill if\kern0.5em y*\kern0.5em \le \kern0.5em {\mu}_1\hfill \\ {}\hfill if\kern0.5em {\mu}_1\kern0.5em \le \kern0.5em y*\kern0.5em \le \kern0.5em {\mu}_2\hfill \\ {}\hfill if\kern0.5em {\mu}_2\kern0.5em \le \kern0.5em y*\hfill \end{array} $$

Then the ordered logit model uses the observations on *y*, which are a form of censored data on y*, to fit the parameter vector *β*. The *μ’*s were unknown parameters that needed to be estimated with *β’*s. Then we could estimate the probabilities of *y* taking different values using the estimated parameters. Here, N = 2, the probability that *y* took on a particular value were found as below:

$$ P\left(y=0\right)\kern0.5em =\kern0.5em \frac{1}{1+ \exp \left(Z-{\mu}_1\right)} $$

$$ P\left(y=1\right)\kern0.5em =\kern0.5em \frac{1}{1+ \exp \left(Z-{\mu}_2\right)}\kern0.5em -\kern0.5em \frac{1}{1+ \exp \left(Z-{\mu}_1\right)} $$

$$ P\left(y=2\right)\kern0.5em =\kern0.5em 1-\frac{1}{1+ \exp \left(Z-{\mu}_2\right)} $$

Now, using the estimated value of *Z* and the assumed logistic distribution of the disturbance term (ε), the ordered logit model could be used to estimate the probability that the unobserved latent variable y* falls within the various threshold limits.

### Analysis

Using an ordered logit model we investigated the factors affecting the willingness to accept fixed income for properly rendering duties of a full-time CHW. The model used *equilibrium* fixed reservation income level as the dependent variable divided into three groups: inactive group: BDT 200–1200 (US$ 2.96-US$ 17.77) per month, moderately active group: BDT 1300–1900 (US$ 19.25-US$ 28.14) per month and active group: BDT 2000 (US$ 29.62) and above per month. We used different socioeconomic, demographic and program-related variables as explanatory variables to test our pre-conceived hypotheses.

Initially, to test our hypotheses we started with all potential factors affecting willingness to accept fixed monthly reservation income. We used simple ordered logistic models to find significant factors. Then we selected factors with *p* value ≤ 0.1 for inclusion in the multiple logistic model. Finally, we selected our model based upon drawing repeated (1000 times) bootstrap samples [24] from the original dataset. In each bootstrap sample, we ran multiple ordered logistic model to find which variables were significant and how many times (Table 1). We found that frequency of significance of coefficients (at 5% level) for the variable of household loan, monthly past income, monthly current income, and ‘joined with an expectation of income’ were more than 90%, whereas it was between 40%-80% for the variables of ‘competition with number of providers’, ‘alternative income generating scope’, VO membership, ‘health training received outside BRAC’ and performance score (see construction of performance score in Alam *et al.* [10]). We found frequency of significance very low (12%-22%) for the demographic characteristics of CHWs along with a variable on whether they had any other income generating involvement. Then we developed a preliminary predictive model with the variables for those frequencies of significance to be independent predictors of outcome were high. We sequentially added other variables to this preliminary model according to the proportion of bootstrap samples in which we selected these variables as significant predictors. Thus we proposed several models with the predictive accuracy of models based on Akaike information criterion (AIC), Bayesian information criterion (BIC), pseudo R square and likelihood ratio test (Table 2). We identified a final model (40%) based on a complicated trade-off between smallest AIC, BIC and larger R square value with the help of likelihood ratio test. We presented results from the final model in Table 3.

**Table 1:** **Number of times a variable significant in 1000 bootstrap simulation, Dhaka urban slums, 2008**

**Explanatory variables**	**Frequency of significance of coefficients (at 5% level)**
Household loan	95.9%
Monthly past CHW income	93.3%
Monthly current CHW income	93.2%
Joined as a CHW with expectation of income	92.2%
Competition with number of other providers	76%
Alternative income generating scope	72.4%
Village organization membership	72.2%
Health training received outside BRAC	56.1%
Performance score	42.2%
Age	20.8%
Other income generating involvement	18.8%
Currently married	12.3%

**Table 2:** **Characteristics of the model, Dhaka urban slums, 2008**

**Percentage**	**Akaike information criterion**	**Bayesian information criterion**	**Pseudo R square**	**Log likelihood**	**LR test ** ***p*** -value*	**# of significant variables**
90%	1056.84	1082.25	0.0545	−522.42	0.36	All of 4
70%	1044.19	1082.30	0.0713	−513.09	0.41	All of 7
40%	1039.14	1085.72	0.0795	−508.57	0.62	All of 9
All	1041.41	1100.69	0.0829	−506.70	0.71	8 of 12

*Approximate likelihood-ratio test of proportionality of odds across response categories.

**Table 3:** **Factors affecting willingness to accept fixed monthly income of CHWs, Dhaka urban slums, 2008**

**Explanatory variables**	**Coefficient** ^**†**^	**Robust Std. error**
Joined as a CHW with expectation of income	0.623**	0.189
Performance score	0.030*	0.016
Monthly current CHW income	0.001**	0.000
Monthly past CHW income	0.001**	0.000
Alternative income generating scope	−0.475**	0.180
Household loan	−0.652**	0.181
Competition with number of other providers	−0.021**	0.008
Village organization membership	0.464**	0.191
Health training received outside BRAC	0.721*	0.366
/cut1	0.198	0.317
/cut2	1.387	0.323

**Significant at 1% level.

*Significant at 5% level.

^†^Coefficients (bias corrected observed bootstrap coefficients).

## Results

We presented socio-demographic characteristics of volunteer CHWs in Table 4. The age of CHWs varied from 16 years to 60 years with a mean age of 32 years. Eighty-six percent of CHWs were currently married although being married was one of the criteria to be a volunteer CHW at BRAC. Around 33% of CHWs had no formal education. We observed a large variation in monthly household income varied from BDT 500 (US$ 7.41) to BDT 63200 (US$ 935.99) with a mean value of BDT 8857 (US$ 131.17).

**Table 4:** **Socio-demographic characteristics of CHWs, Dhaka urban slums, 2008**

**Characteristics**	**Mean/%**	**Standard deviation**	**Minimum**	**Maximum**
Age	32.24	9.10	16	60
Currently married	86.08%	-	0	1
No education	33.73%	-	0	1
Monthly household income of CHWs (US$)	131.17	99.20	7.41	935.99
Wealth score	−0.06	1.00	−1.85	2.19
Village organization membership	35.49%	-	0	1
Household loan	42.94%	-	0	1
Length of CHW experience	14.19	3.73	3	22
Health training received outside BRAC	7.84%	-	0	1
Joined as a CHW with expectation of income	60.98%	-	0	1
Performance score	10.69	5.93	0	20
Monthly current CHW income (US$)	8.21	5.81	0.74	59.24
Positive family attitude	81.96%	-	0	1
Community approval	9%	-	0	1
Social prestige	15.95	0.89	−2.24	1.32
Competition with number of other providers	19.72	11.28	0	68
Other income generating involvement	37.84%	-	0	1
Alternative income generating scope	45.49	-	0	1
Monthly past CHW income (US$)	13.40	21.48	0	222.15

*US$ 1 = 67.52 BDT (1^st^ July 2008).

**Figure 1:**
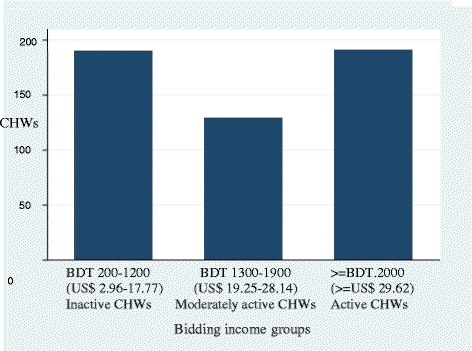
**Bidding income level of BRAC female volunteer CHWs, Dhaka urban slums, 2008.**

The average reservation wage of CHWs was BDT 1628 (US$ 24.11). Around 37% of CHWs asked for fixed income of BDT 200–1200 (US$ 2.96-US$ 17.77), 25% for BDT 1300–1900 (US$ 19.25-US$ 28.14) and 37% for BDT 2000 (US$ 29.62) and above (Figure 1).

The result suggests that CHW who started their work with an expectation of income were 62% more likely (significant at 1% level) to ask for higher reservation wage compared to those who joined CHW work for other reasons. CHWs who were performing their duties at higher level were 3% more likely (significant at 5% level) to ask for reservation wage compared to those who were not performing at that level. We also found that CHWs with monthly higher current income and higher past income were more likely (significant at 1% level) to expect higher reservation wage. On the contrary, CHWs who had alternative income generating scope were 47% less likely (significant at 1% level) to ask for higher reservation wage. Likewise, CHWs who were burdened with household loan were 65% less likely (significant at 1% level) to ask for higher reservation wage. Similarly, CHWs who were facing competition with other health services providers were 2% less likely (significant at 1% level) to ask for higher reservation wage. But CHWs with VO membership were 46% more likely (significant at 1% level) to ask for higher reservation wage compared to those who were not VO members. Finally, CHWs who received additional health training outside BRAC were 72% more likely (significant at 5% level) to ask for higher reservation wage compared to those who had no such additional health training (Table 3).

**Figure 2:**
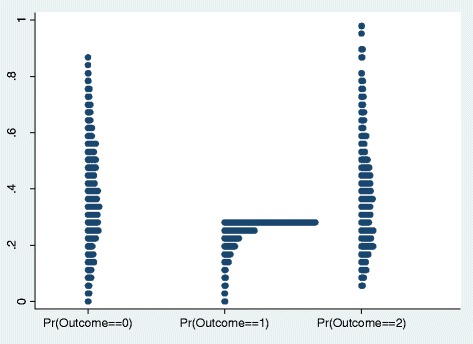
**Predicted values of three outcomes (inactive, moderately active and active BRAC female volunteer CHWs), Dhaka urban slums, 2008.**

Table 5 shows marginal effect of the explanatory variables of our model. The results suggest that marginal effect of almost all the explanatory variables in the model had a lower magnitude for the reservation wage of moderately active CHW group whereas it had higher magnitude for the reservation wage of inactive and active CHWs groups. It might imply that a larger fraction of variations in wage level could be explained by the explanatory variables in higher and lower level of fixed reservation wage ranges. We found the effect of outside health training the strongest in absolute value and it increased the likelihood to be in the highest reservation wage group by 18.1% (significant at 5% level) but decreased the likelihood to be in the lowest reservation group by 15% (significant at 5% level). We observed similar trends for the variable of ‘VO membership’ (11% vs.10.4%, significant at 5% level) and ‘joined as a CHW with an expectation of income’ (14.6% vs.15%, significant at 5% level). However, it also suggests that household loan and alternative income generating scope reduced the likelihood to be in the highest reservation wage group by 15.1% and 10.9% (significant at 5% level) respectively. Figure 2 depicts the predictions of the occurrence of outcomes with special attention to outcome 1 (moderately active CHW group) which is more likely the average choice of *Manoshi* CHWs.

**Table 5:** **Marginal Effects for the covariates in the final ordered logit model, Dhaka urban slums, 2008**

**Explanatory variables**	**Outcome 0 (Inactive)**	**Outcome 1 (Moderately active)**	**Outcome 2 (Active)**
Joined as a CHW with expectation of income	−0.150*	0.004	0.146*
Performance score	−0.007*	0.000	0.007*
Monthly current CHW income	0.000*	0.000	0.000*
Monthly past CHW income	0.000*	0.000	0.000*
Alternative income generating scope	0.109*	0.000	−0.109*
Household loan	0.153*	−0.002	−0.151*
Competition with number of other providers	0.005	0.000	−0.005
Village organization member	−0.104*	−0.006	0.110*
Health training received outside BRAC	−0.150*	−0.031	0.181*
Xmfx_y	0.350	0.289	0.361

**Significant at 1% level.

*Significant at 5% level.

## Discussion

The result demonstrated that monthly average reservation wage of CHWs to continue their work as full-time CHWs rather than volunteer CHWs was three times higher than the monthly average current income of US$ 8.03 reported elsewhere [10]. One of the possible reasons that spurred their expected level of income is that though they would be in the same job their total working hours would be increased. They would be required to serve as full-time workers with only a day-off what their other field-based colleagues used to enjoy. The flexible working hours that they are enjoying as volunteers would not be available any more and this certainly would increase their opportunity cost to be in the labour market. A number of studies suggests that reservation wage for women largely depends on their family duties that are assigned socially [25–27], and this finding is relevant for the BRAC female volunteer CHWs. Besides, their freedom would be furthermore impeded as their work would be closely monitored by their supervisors. According to *stress-hypothesis*, heavy workload combined with low decision latitude gives cause for stress [28]. This might be another reason of asking for a higher compensation by BRAC CHWs under the proposed hypothetical conditions.

The study has captured both financial and non-financial factors as important predictors of fixed reservation wage of BRAC female volunteer CHWs. Despite the explicit role of financial incentives in increasing retention and performance of BRAC volunteer CHWs the assumption of constant non-financial incentives is relevant to our results in a sense that the association of BRAC always offers volunteer CHWs a range of common non-financial incentives irrespective of variations in performance-based financial incentives over time. Our current analysis has found financial incentives i.e., CHW’s joining BRAC with an expectation of income, performance score (higher performance is indicative of higher financial incentives in a performance-based incentive system), and monthly current and past CHW income as positive predictors of fixed reservation wage. On the other hand, the study found non-financial incentives i.e., alternative income generating scope, burden of household loan, competition with number of other providers, VO membership, and additional health training received outside BRAC as significant predictors of fixed reservation wage.

We found that CHWs who joined with an expectation of income rather than serving the community were more likely to ask for higher reservation wage. The possible reason of asking for higher reservation wage might be that CHWs would like to maximize their *utility* from the financial incentives rather than other non-financial enticement attributed to this offer. A recent study observed similar findings that those CHWs joined BRAC with an expectation of income were more likely to continue their job compared to other CHWs [9].

Factors that are indicative of the performance of CHWs also determine the level of their reservation wage. One of the direct indicators that we used in our model was performance score of CHWs in which they had been assessed on a composite score based on four core activities described elsewhere [10]. Besides, monthly income of CHWs could be considered as an indirect indicator of the performance of CHWs since such income largely depends on their activities. Therefore, an active CHW was more likely to have a higher level of monthly income. The result suggests that CHWs who are performing well would like to continue their work if they would have been compensated with higher level of reservation wage. Besides current income, we also found past income of CHWs an important determinant of their reservation wage. It also indicated that CHWs who had experience of doing other work in the past or entered in the labour market for a longer period of time would ask for higher reservation wage. Other studies also have confirmed that work experience has a positive association with the level of reservation wage [19,29,30].

On the other hand, the likelihood of asking for higher reservation wage decreased for the CHWs who had alternative income generating scope. One explanation might be that these CHWs compromised financial income from CHW activities with the social prestige and community approval they used to enjoy because of the association with BRAC, and they might use the ‘good name’ of BRAC for earning from alternative sources. The other explanation might be that the current flexible volunteer work arrangements along with other income generating opportunities might be a better-fit for BRAC volunteer CHWs than the proposed restricted conditions on which the bidding negotiation took place.

Besides, non-tangible assets such as education attainment that increases the qualification of a person might be important in determining the reservation wage. It was found that person with a higher education is more likely to ask for higher level of reservation wage [31]. In our study, most of the CHWs were without any formal education or with primary education only and we didn’t observe any significant association between their education attainment and reservation wage. However, in the current study we found that CHWs with additional health training received outside BRAC significantly asked for higher reservation wage. In BRAC all CHWs receive same basic and refresher training from BRAC but additional training received outside BRAC makes a difference among them. The additional training boosts up their confidence level and makes them feel to be more positive regarding their aptitude for the CHW activities. It is also reflected in the reservation wage that what they were asking for was significantly higher for the CHWs with additional health training received outside BRAC compared to those who did not receive any such additional training other than the basic and refresher training from BRAC. Previously, additional health training received outside BRAC was also found to be an important determinant for the performance of volunteer CHWs [14].

We also found that VO membership of CHWs as microfinance borrowers from BRAC increased the likelihood of asking for higher reservation wage. One of the possible reasons might be that they were in better position in terms of bargaining power since they were more closely attached to BRAC compared to the CHWs who were not VO members. Rahman & Tasneem [14] found that CHWs who were VO members were more likely to perform better and earn more compared to other non-VO member CHWs. However, liability of the household negatively affected the level of reservation wage of CHWs. CHWs who were burdened with credit were ready to accept job offer at a lower wage level. It is a common notion that individuals with credit burden are in a vulnerable situation and does not hold enough bargaining power to negotiate on higher compensation. Similarly, we found that the CHWs who faced competition from other health services providers were less likely to ask for higher reservation wage. It is our interpretation that competition from other health services providers threatened their potential income earning from CHW activities and put them in a position with less bargaining power.

A number of studies found financial asset to be an important determinant of reservation wage [19,29,32,33]. The wealthier people can afford to stay unemployed for a longer period of time due to their better economic status [34]. In our study we used asset holdings of the CHW households to see whether it affected the level of their reservation wage. But we didn’t find any significant association between asset holdings and the reservation wage. One of the possible reasons might be there was very insignificant differences among the CHWs in terms of their wealth status since all of them were slum dwellers with similar kind of asset holdings.

The study has several limitations which one needs to consider. Other non-financial factors such as positive family attitude, community approval, positive community appraisal, involvement with other NGOs, change in social prestige and expectation of social recognition were found to be important predictors of retention and performance in past studies [9–11]. However, the current modelling is not able to capture these non-financial factors. Additional efforts along with more specific methodological approaches need to be employed to capture the contribution of these non-financial factors. Besides, there are a number of other important factors as suggested by past studies such as knowledge of the worker about the current labour market, number of other employed persons in the household, job security, and family responsibility that we could not consider for our model [19,20,35]. Moreover, understanding the determinants of the reservation wage of the CHWs who had already dropped out from BRAC would be interesting and could be explored in future.

## Conclusions

The results of the bidding game exercise have implications both in improving retention of volunteer CHWs as well as their level of performance. The findings of this study are relevant to low-income countries, such as Bangladesh and Tanzania, which frequently employ volunteer CHWs in community based health programs, and high dropout and poor level of performance of CHWs are common problems there due to their erratic and performance-based income. The underlying results of the current study could positively influence retention and level of performance of volunteer CHWs by promoting fixed reservation income for them. So, the implementation of the findings of current study could potentially address the shortage of trained health workers as well as their poor level of performance in these low-income countries by retaining trained CHWs and motivating them for higher level of performance. This could contribute to effective and viable community health programs in low-income countries and thus could accelerate the attainment of the health-related MDGs by the stipulated time frame.

In the current study, factors influencing the fixed reservation wage positively included additional health training received outside BRAC, ‘joined as a CHW with an expectation of income’, and BRAC VO membership. Negative correlates were outstanding loan commitments of household and alternative income generating scope of CHWs. The findings of this study clearly revealed that reservation wage was significantly higher for the CHWs who were well-performing and had better qualifications related to the health service delivery. These are the CHWs who need to be retained for increasing the effectiveness and sustainability of the community based health programs implemented by BRAC and others.

Currently BRAC CHWs receive a benefit lower than the reservation wage, but have considerable flexibility in their working conditions, and work is not rigorously monitored. If working conditions become more demanding and monitoring becomes stricter, it is important for the program to provide an optimal system of incentives, and these should generally be higher than the reservation wage, to ensure a sustainable, high performing and motivated community health workforce.

Therefore, the present study suggests that the financial incentives provided to CHWs should be clearly based on their qualifications and opportunity cost; otherwise, poor retention and performance cannot be prevented. However, considerable time and effort may be needed to develop an optimum model of measuring and monitoring these factors. This effort is considered to be well spent if it results in an effective incentive system for the volunteer CHWs based on these criteria in future.
